# Dataset for investigating triacylglycerol accumulation in PBCV-1 infected *Chlorella*

**DOI:** 10.1016/j.dib.2025.112220

**Published:** 2025-10-28

**Authors:** Amanda M. Lopez, Yoonjung Choi, Zhi Zhou

**Affiliations:** a School of Sustainability Engineering and Environmental Engineering, Purdue University, West Lafayette, IN 47907, United States; b Department of Agricultural and Biological Engineering, Purdue University, West Lafayette, IN 47907, United States; c Lyles School of Civil and Construction Engineering, Purdue University, West Lafayette, IN 47907, United States

**Keywords:** Biofuel, Chlorella, Unsupervised machine learning, Single-cell, Lipids, Viral infection

## Abstract

*Chlorella* is a promising biofuel source due to its high lipid accumulation, rapid growth, and suitability for inland cultivation. However, how the *Paramecium bursaria Chlorella* virus 1 (PBCV-1) influences its triacylglycerol (TAG) accumulation remains underexplored. This data article provides a detailed description of the dataset generated to investigate TAG accumulation profiles in *Chlorella* infected with PBCV-1. The data, collected via high-resolution epifluorescence microscopy of over 4000 single cells across a full lytic cycle, includes measurements of TAG accumulation, chlorophyll fluorescence, and nuclear morphology, along with extracellular nutrient concentrations to rule out nutrient stress as a confounding factor. This dataset can be reused by researchers to develop new image analysis algorithms, train machine learning models, investigate virus-host interactions, and inform the development of more cost-effective biofuel production strategies.

Specifications Table

**Table:** 

Subject	Biology
Specific subject area	Biofuel
Type of data	Table (.csv format)
Data collection	To understand how cells respond to PBCV-1 infection, six algal samples (three infected with PBCV-1 and three uninfected controls) were collected at 0, 2, 6, 12, 18, and 24 h post-infection for high-resolution epifluorescence microscopy. Neutral lipids were stained with BODIPY® 505/515 and DAPI was used to define nuclear contours, a method adapted and optimized from microalgae analysis. Images were captured on a Nikon Eclipse Ni-U microscope with fixed exposure times across DAPI, BODIPY, and chlorophyll channels to ensure consistent comparisons of fluorescence intensities. NIS-Elements D software was used for acquisition and Fiji (ImageJ 1.54f) for quantitative analysis, extracting cell area, TAG, and chlorophyll intensities. Extracellular nutrient data were collected to rule out nutrient stress as a confounding factor. Nitrate (NO_3_^–^) and phosphate (PO_4_^3-^) concentrations at each time point were quantified using ion chromatography (IC) on a Metrohm 940 Professional IC Vario system. An anion eluent composed of 3.2 mM sodium carbonate and 1 mM sodium bicarbonate was employed for the separation of anionic species through a Metrosep A Supp 5–150/4.0 anion exchange column operated at a flow rate of 0.7 mL min^-1^ and maintained at 30 °C. Calibration curves were prepared using a certified anion standard mix (Metrohm Anion Mix 3) and spanned a concentration range from 0.1 mg l^-1^ to 100 mg l^-1^ (R^2^ >0.999).
Data source location	Purdue University, West Lafayette, Indiana, United States
Data accessibility	Repository name: Zenodo Data identification number: 10.5281/zenodo.17399774 Direct URL to data: https://zenodo.org/records/17399774
Related research article	Enhanced biomimetic algal lipid enrichment for improved biofuel production driven by non-stress viral lysis

## 1 Value of the Data

• This dataset offers insights into the intricate relationship between *Chlorella* sp. and the lytic virus PBCV-1, focusing on how viral infection impacts the host's TAG accumulation. The data capture phenotypic dynamics throughout a complete viral lytic cycle at a multiplicity of infection (MOI) of 9.2 × 10^−6^ viral particles per algal cell, during which 82.4 % of Chlorella cells were infected by 24 h. Infection efficiencies of PBCV-1 vary depending on experimental conditions and MOI. Based on prior research [3], all PBCV-1 MOI from 1.46 × 10^–8^ to 1.46 × 10^–2^ resulted in complete *Chlorella* cell disruption in <142 h.
• A standout feature of this dataset is its high-resolution epifluorescence microscopy analysis of over 4000 single cells. Unlike bulk measurements, which provide averaged population responses, single-cell data uncovers the inherent complexity of infection dynamics, revealing cell-to-cell variability and transient physiological states that are otherwise overlooked. Such heterogeneity is central to understanding how subpopulations within a culture respond differently to viral stress, influencing overall culture stability, resilience, and productivity. Through the capture of these fine-scale fluctuations in TAG accumulation, chlorophyll fluorescence, and nuclear morphology during the infection cycle, this dataset connects fundamental cell biology with the applied goals of algal bioprocesses.
• The practical applications of this dataset are significant for both biotechnology and computational biology. For biofuel research, understanding how viral infections influence lipid biosynthesis in *Chlorella* can directly inform the development of more efficient and cost-effective strategies for algal cultivation and lipid extraction. Concurrently, the rich, quantitative imaging data provides a foundation for computer scientists and bioinformaticians to develop and train sophisticated image analysis algorithms and machine learning models, which can automate and enhance the characterization of microalgal cellular processes.

## 2 Background

*Chlorella* is a freshwater, non-calcifying microalgal genus and is considered a promising candidate for biofuel production due to its rapid biomass growth, lipid accumulation capability, and suitability for scalable inland cultivation [1]. Despite its potential, lipid yields are typically low (<0.018 g l^-1^ d^-1^) and primarily enhanced under abiotic stressors such as nutrient limitation, high light intensity, or salinity [2]. However, the role of biotic stressors, such as algal viruses, remains comparatively understudied. In algal cultivation systems like open ponds or large-scale photobioreactors, *Chlorella* populations are frequently exposed to Paramecium bursaria Chlorella virus 1 (PBCV-1), a large dsDNA virus known to hijack the host’s metabolic machinery and profoundly reprogram its cellular pathways to favor viral genome replication [3]. Despite this extensive cellular takeover, the effects of PBCV-1 infection on *Chlorella* lipid metabolism, especially in the synthesis of triacylglycerols (TAGs), remain uncharacterized. This represents a significant knowledge gap in how viral infection may reshape host lipid fluxes and metabolite pools relevant to biofuel applications. To address this, phenotypic data were collected at the single-cell level using high-resolution epifluorescence microscopy. Single-cell analysis provides key advantages over conventional bulk approaches: (i) it enables non-destructive monitoring of TAGs droplets formation within individual cells, (ii) it uncovers transient and heterogeneous metabolic shifts that are otherwise masked in bulk averages, and (iii) it differentiates TAGs from structural polar lipids, which bulk gravimetric methods cannot effectively resolve.

## 3 Data Description

The raw data in .csv file format contains seven types of variables and data from each of the 4000 single cells analyzed. The variables are cell type (positive control, negative control, or PBCV1-Chlorella), hour post infection (hpi), cell area (µm^2^), mean lipids-bodipy fluorescence intensity per cell (arbitrary fluorescence units (AFU)/µm^2^), total lipids-bodipy fluorescence intensity per cell (AFU), mean chlorophyll fluorescence intensity per cell (AFU/µm^2^), and total chlorophyll fluorescence intensity per cell (AFU). The data were used with multivariate unsupervised machine learning to evaluate the enhanced biomimetic algal lipid enrichment for improved biofuel production driven by non-stress viral lysis[4]. Detailed data are deposited in Zenodo[5].

## 4 Experimental Design, Materials and Methods

**Microalgae strain and culture conditions.** The freshwater microalgal strain used in this study was *Chlorella* sp. (ATCC 50,258), acquired directly from the ATCC (American Type Culture Collection). This particular strain was chosen due to its well-established role as a host for the lytic virus PBCV-1 (*Paramecium bursaria* chlorella virus 1), making it ideal for investigating virus-host interactions. Stock cultures of *Chlorella* sp. were maintained under controlled conditions to ensure consistent growth and physiological states. The cultures were kept at a constant temperature of 25∘C and subjected to a regulated 14-hour light/10-hour dark photoperiod. Illumination was provided by a 25 W full-spectrum grow light, which delivered an optimized level of photosynthetically active radiation (PAR) to the culture surface, promoting efficient photosynthesis. To prevent cell settling and ensure uniform nutrient and gas distribution throughout the culture, a continuous agitation rate of 125 rpm was maintained. This constant mixing also facilitated optimal gas exchange, crucial for algal respiration and growth. Algal growth occurred in ATCC medium 847, a specialized and nutrient-rich formulation designed to support robust Chlorella proliferation. The medium's composition includes essential components such as proteose peptone, sodium nitrate, calcium chloride, magnesium sulfate, and a combination of dibasic and monobasic potassium phosphate. Additionally, sodium chloride and ferric chloride solution were included, providing necessary micronutrients and maintaining osmotic balance for healthy cellular function.

**Experimental setup and viral infection dynamics.** A total of 300 mL of *Chlorella* sp. culture, in its early exponential growth phase, was divided into six sterile 50 mL polypropylene conical-bottom tubes, with 50 mL allocated to each tube. For the experimental treatment, three of these tubes received an inoculation of PBCV-1, while the remaining three tubes were designated as uninfected controls. This setup ensured the acquisition of triplicate biological samples for both the infected and control conditions. Throughout the experiment, all cultures were maintained under rigorously identical abiotic conditions to guarantee direct comparability of results. Viral inoculation was performed one hour after the commencement of the light period. The inoculation involved the addition of viral lysate to the algal culture at a volumetric ratio of 1:167. This ratio resulted in a MOI of 9.2 × 10^−6^ viral particles per algal cell. This deliberately low MOI was selected based on prior research[6], which indicated that even significant variations in MOI led to only minor alterations in the overall lytic cycle duration. The implementation of a low MOI in this study served to extend the early and mid-infection stages, thereby facilitating a more comprehensive and detailed characterization of the intracellular metabolic shifts occurring within the Chlorella cells. Furthermore, viral infection efficiency was quantitatively assessed at each designated time point by calculating the ratio of infected cells to the total number of viable cells present at 0 h hpi.

**Sample preparation and staining for microscopy.** To capture the dynamic cellular responses of Chlorella to PBCV-1 infection, samples were systematically harvested at predetermined time points: 0, 2, 6, 12, 18, and 24 hpi. These samples were then prepared for high-resolution in vitro epifluorescence microscopy. For visualization of cellular components, neutral lipids, primarily representing TAGs, were stained using BODIPY® 505/515 (Invitrogen) at a concentration of 100 mg mL^−1^. Simultaneously, nuclear contours were delineated for subsequent single-cell segmentation by staining with 4′,6-diamidino-2-phenylindole (DAPI) (Sigma-Aldrich) at 2 mg mL^−1^. To ensure consistent and thorough labeling across all samples, 1 mL of each stain was added to 200 µL of the Chlorella culture at each time point. The mixtures were briefly vortexed and then incubated in the dark for 15 min. This single-cell staining methodology was carefully adapted and optimized from a previously established framework designed for microalgae analysis[7].

**Image acquisition and quantitative analysis.** Image analysis was performed using a Nikon Eclipse Ni-U upright microscope, which was outfitted with a mercury arc lamp for fluorescence excitation, a high-magnification 40x objective lens, and a DS-Qi2 camera for image capture. To enable robust and comparable quantification of fluorescence intensities across all samples and time points, fixed exposure times of 1 s were uniformly applied for all three fluorescence channels: DAPI (UV excitation, 460 nm emission), BODIPY (FITC excitation, 500 nm emission), and chlorophyll autofluorescence (Cy5 excitation, 670 nm emission). Images were systematically acquired utilizing NIS-Elements D software and subsequently subjected to quantitative analysis using Fiji (ImageJ 1.54f). Cell outlines, identified by their DAPI fluorescence, served as the basis for accurate extraction of key cellular parameters, including cell area, and the intensities of TAGs and chlorophyll (quantified in arbitrary fluorescence units (AFU)). For the internal validation of the fluorescence signals, *Escherichia coli* was strategically included as a negative control, leveraging its inherent lack of both TAGs and chlorophyll[8], thus providing a clear baseline for background fluorescence.

## Limitations

A primary limitation of this study, common across most biological systems, stemmed from the inherent heterogeneity of cellular populations and the diverse biological responses observed, even among replicate samples, under low MOI conditions. While a metabolically reprogrammed subpopulation, enriched in TAG fluorescence, was successfully identified, the asynchronous nature of PBCV-1 infection introduced unavoidable variability. It's highly probable that not all infected cells followed identical metabolic trajectories, suggesting that the emergence of distinct subgroups necessitates additional, more temporal resolution in future investigations.

## Ethics Statement

The authors have read and follow the ethical requirements for publication in Data in Brief and confirm that the current work does not involve human subjects, animal experiments, or any data collected from social media platforms.

## CRediT authorship contribution statement

**Amanda M. Lopez:** Data curation, Formal analysis, Investigation, Methodology, Visualization, Writing – original draft. **Yoonjung Choi:** Investigation, Validation. **Zhi Zhou:** Conceptualization, Funding acquisition, Project administration, Supervision, Writing – review & editing.

## Data Availability

zenodoDataset for Understanding PBCV-1 Influence on Chlorella Lipid Metabolism and Biofuel Potential (Original data).
